# A Novel Algorithm for Forensic Identification Using Geometric Cranial Patterns in Digital Lateral Cephalometric Radiographs in Forensic Dentistry

**DOI:** 10.3390/diagnostics14171840

**Published:** 2024-08-23

**Authors:** Shahab Kavousinejad, Mohsen Yazdanian, Mohammad Mahboob Kanafi, Elahe Tahmasebi

**Affiliations:** 1 Research Center for Prevention of Oral and Dental Diseases, Baqiyatallah University of Medical Sciences, Tehran 1435916471, Iran; shahabkavousinejad@sbmu.ac.ir (S.K.); myazdaniandr@gmail.com (M.Y.); 2 School of Dentistry, Baqiyatallah University of Medical Sciences, Tehran 1435916471, Iran; 3 Human Genetics Research Centre, Baqiyatallah University of Medical Science, Tehran 1435916471, Iran; mm.kanafi@yahoo.com

**Keywords:** forensic dentistry, identification, lateral cephalogram, algorithm

## Abstract

Lateral cephalometric radiographs are crucial in dentistry and orthodontics for diagnosis and treatment planning. However, their use in forensic identification, especially with burned bodies or in mass disasters, is challenging. AM (antemortem) and PM (postmortem) radiographs can be compared for identification. This study introduces and evaluates a novel algorithm for extracting cranial patterns from digital lateral cephalometric radiographs for identification purposes. Due to the unavailability of AM cephalograms from deceased individuals, the algorithm was tested using pre- and post-treatment cephalograms of living individuals from an orthodontic archive, considered as AM and PM data. The proposed algorithm encodes cranial patterns into a database for future identification. It matches PM cephalograms with AM records, accurately identifying individuals by comparing cranial features. The algorithm achieved an accuracy of 97.5%, a sensitivity of 97.7%, and a specificity of 95.2%, correctly identifying 350 out of 358 cases. The mean similarity score improved from 91.02% to 98.10% after applying the Automatic Error Reduction (AER) function. Intra-observer error analysis showed an average Euclidean distance of 3.07 pixels (SD = 0.73) for repeated landmark selections. The proposed algorithm shows promise for identity recognition based on cranial patterns and could be enhanced with artificial intelligence (AI) algorithms in future studies.

## 1. Introduction

Radiographic analysis has long been utilized in various medical disciplines to gain valuable insights into internal structures and aid in diagnosis. Cranial radiography includes posterior–anterior (frontal) and lateral cephalometric X-rays, as well as other views such as the submentovertex and occipitomental projections (Waters’ view). Cephalometric analysis is utilized in orthodontics and maxillofacial surgery to diagnose and address potential abnormalities in the jaws and teeth [1]. This analysis involves measuring both angles and distances. By identifying reference points in the craniofacial skeleton and creating reference lines, we can measure the linear distances and angles between these lines. Comparing these measurements to normal values helps detect skeletal and dental anomalies [2]. Other applications include predicting growth through cervical vertebra analysis [3], evaluating pituitary tumors [4] (such as sella turcica enlargement), and monitoring changes from orthodontic and orthognathic surgery treatments [5].

In addition to these benefits, it can also be employed in the identification of bodies, particularly in cases where soft tissue has been completely lost [6]. However, when it comes to forensic identification, accurately determining the identity of individuals based solely on radiographs presents a significant challenge. In such complex scenarios, where traditional identification methods, such as fingerprinting or DNA analysis, may simply not be feasible, the importance of using skeletal [7], hard tissue [8], and dental structures [9] for identity verification becomes evident [10]. This is performed by comparing antemortem (AM) and postmortem (PM) data such as radiographs [11,12].

Cranial hard tissue offers valuable information that can aid in identification purposes [13]. The cranial bones possess unique features and characteristics, including the shape and structure of the skull [14], which can provide clues about an individual’s identity [13]. These characteristics develop throughout an individual’s growth and development, influenced by a complex interplay between genetic and environmental factors [15]. Genetic and environmental factors [16] interact to influence cranial growth and development, leading to variations in the shape and geometric dimensions of the cranium among different individuals [17,18]. The cranial base matures around 7–8 years of age, followed by the cranial floor at 11–12 years of age (up to 15 years of age [19]), the neurocranium at 9–10 years of age, and the maxillomandibular structures at 15–16 years of age [14]. It is reported that male and female skulls differ anatomically, which is useful for gender identification [20].

Extensive research has demonstrated the utility of the frontal sinus as a valuable feature for human identification [21,22] and differentiating gender [23]. The frontal sinus is one of four pairs of paranasal sinuses, comprising asymmetrical, air-filled cavities located in the anterior region of the frontal bone. It serves to reduce the weight of the skull and protect the brain from injury. The formation of the frontal sinus begins around the fourth or fifth month of fetal development, with active growth occurring by the age of two or three [24,25]. It becomes radiographically visible by four or five years of age and continues to develop and morphologically change throughout puberty [24,25]. Typically, its growth is complete by age 20 [26], after which it remains stable in adulthood, unless affected by trauma, chronic sinus disease, or tumors [25]. Yoshino et al. [27] developed a classification system to assess the anatomical uniqueness of frontal sinuses, considering factors such as size, shape, asymmetry, and the presence of additional structures. Their findings identified over 20,000 possible variations, demonstrating the significant potential of frontal sinuses for personal identification. Various studies have explored identity verification based on radiological imaging. Beaini et al. [28] reported that using computed tomography (CT) scans allowed for the accurate segmentation and 3D reconstruction of frontal sinus volumes. In their pilot study involving 20 cone-beam CT scans, they demonstrated that 3D models of the frontal sinuses could be reliably matched to the same individual. Successful identification based on frontal sinuses has been achieved by comparing AM radiographs with PM radiographs [29,30,31]. Gómez et al. [32] introduced an AI-based framework for automating forensic comparative radiography, focusing on frontal sinuses. Their system includes segmentation, superposition, and decision-making methods, validating high-quality segmentation and achieving the automatic shortlisting of 40% of candidates.

However, it is noteworthy that the accuracy of using only frontal sinuses for identification may be relatively lower than considering the entire cranial structure in radiographs. Therefore, incorporating all cranial patterns into the identity verification process may significantly enhance accuracy. Cranial structures possess the potential to represent an individual’s unique identity, such as fingerprints or faces [33]. Therefore, achieving a higher accuracy rate in the context of identity verification is of paramount importance. The mandible is the largest facial bone, and its shape and features, including the gonial angle and mandibular canal, vary by age and gender [34]. Albalawi et al. [35] found that the angle between the gonion and menton, along with ramus dimensions, effectively indicates sexual dimorphism and is useful for sex determination in dental and medicolegal contexts. Bozkurt and Karagol [36] developed a fully automated method for segmenting jaws and teeth in panoramic dental radiographs (OPGs). Their approach achieved high accuracy, with a jaw separation ratio of 0.99 and detection rates of 0.90 for mandibular and 0.92 for maxillary teeth, demonstrating strong potential for automatic human identification. They suggested evaluating several features on lateral skull radiographs, including bigonial width, cranial height, bimaxillary breadth, and other facial measurements. Comparing these features in AM and PM records can provide valuable forensic information [37]. Frontal radiography (PA cephalometric analysis) [37] and cone-beam computed tomography (CT) [38] are both used in forensic identification. PA cephalometric analysis, using Caldwell’s or Waters’ view, assesses frontal sinus variations [39]. Frontal sinus variables include sinus area, height, and width measurements. Cone-beam CT provides 3D images of teeth and surrounding structures, aiding in comparison between AM and PM data, though artifacts from dental restorations can complicate analysis [40]. Although CBCT provides greater accuracy for identification due to its three-dimensional nature, it may not be cost-effective as AM evidence or for PM comparisons.

Several identification methods have been introduced in forensic dentistry, including the analysis of the frontal sinus, age estimation using wrist radiography and OPGs, and identification based on dental characteristics in OPGs, such as restorations, caries, and other features [41]. In previous studies, the use of cranial features in lateral cephalometric radiographs has received less attention. Existing research primarily focuses on frontal sinus features or dental structures, whereas a more comprehensive analysis of cranial features could provide more useful information for identity verification. Moreover, previous studies have not introduced a method (algorithm or software) for identity recognition using cranial skeletal structures in lateral cephalometric radiographs. The algorithm presented in this study introduces a novel approach by automatically extracting and analyzing cranial patterns from lateral cephalometric images. This method aims to address the existing limitations in current identification techniques and has the potential to enhance the accuracy of identity verification. This study presents a novel algorithm for biometric identification, using cranial landmarks in 2D lateral cephalometric radiographs (AM and PM). The algorithm extracts cranial patterns from AM radiographs, encodes them into a database, and utilizes this information for victim identification using PM radiographs. We hypothesized the following: (I) cranial patterns can be extracted from AM lateral cephalometric radiographs and stored in a database; (II) cranial patterns extracted from PM radiographs can be compared with the stored AM patterns to identify the best match; and (III) this process of comparing AM and PM patterns will enable the accurate identification of individuals.

## 2. Materials and Methods

In this study, a new algorithm called the K-Victim Identification Network (K-VIN) was designed and developed for identity recognition using cranial patterns in digital lateral cephalograms. The K-VIN algorithm aims to store AM cranial structural patterns in a database and, in the event of mass casualties such as a fire incident, match PM cranial structural patterns with the available cases in the database to identify the closest matches.

### 2.1. The Proposed K-VIN Algorithm

**Figure 1:**
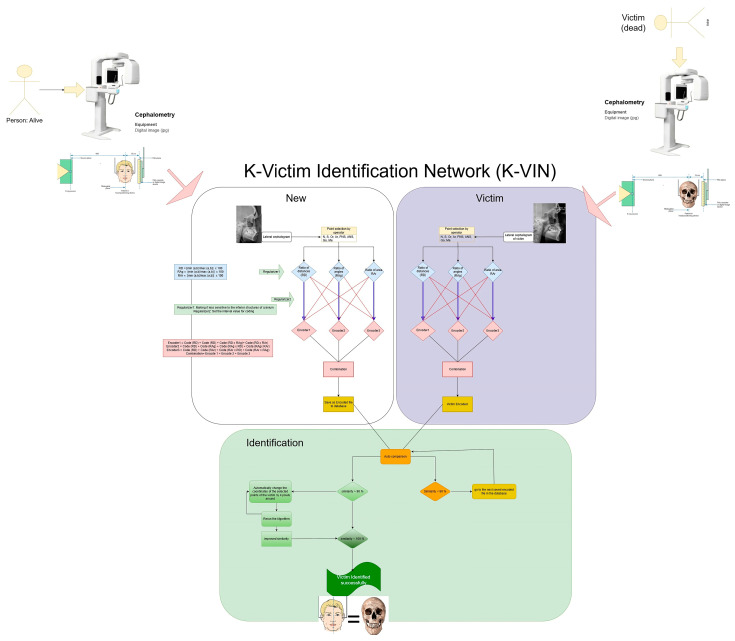
The proposed algorithm.

Figure 1 illustrates the K-VIN algorithm, which comprises three parts: new, victim, and identification. For AM radiographs (new), the process involves selecting landmarks, extracting quantitative features, calculating ratios, encoding the data, and storing them in the database. For PM radiographs (victim), the steps include selecting landmarks, extracting quantitative features, calculating ratios, and encoding the data. The final part, identification, involves auto-comparison. Only the selection of landmarks is manual; all other processes are automated.

### 2.2. Radiographic Landmark Selection for Encoding Cranial AM Structural Patterns

**Figure 2:**
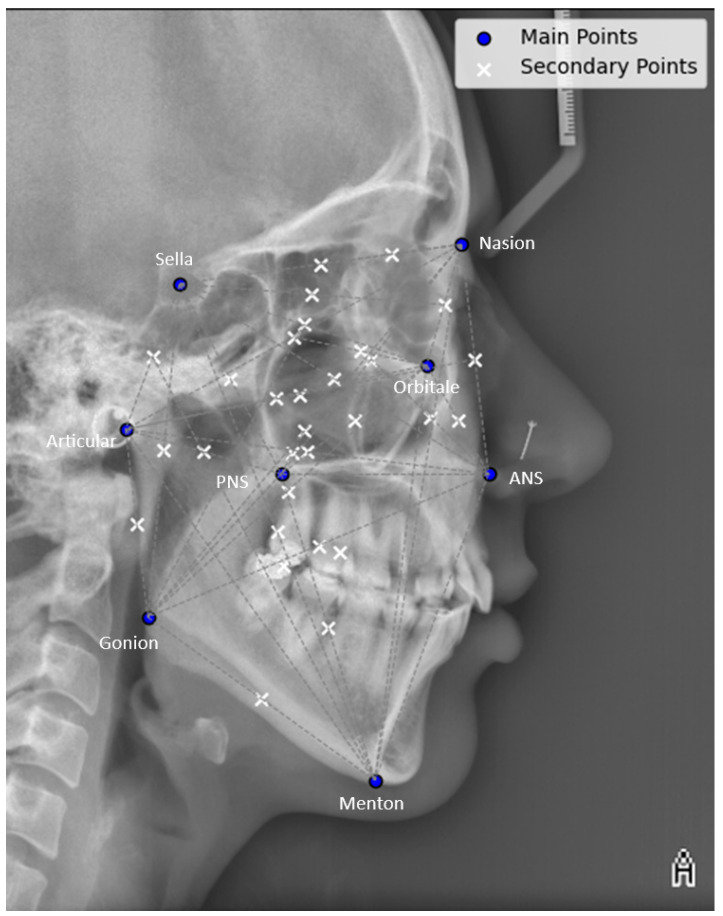
Multiple lines are drawn between the main landmarks (Na, S, Or, Ar, ANS, PNS, Go, and Me) and the secondary landmarks. This set of these points generates numerous distances, angles, and triangles within the cranial region. By defining these features, it is possible to extract individual identification characteristics. More geometric features are calculated in the upper regions compared to the lower regions (mandible). The ratio of features between the upper and lower regions can be customized by the user.

During this stage, the craniofacial bone structure patterns of individuals in the AM phase are encoded using lateral cephalograms. These cephalograms provide valuable information for identification. In this algorithm, eight key landmarks were defined. These landmarks are routinely used in cephalometric analysis within orthodontics and maxillofacial surgery to diagnose skeletal abnormalities and create treatment plans. To use this algorithm, the practitioner first manually determines the landmarks, while the remaining processes are carried out automatically. The user (practitioner), typically an orthodontist, selects the key landmarks in the cranial region (Figure 2) in a specified order [42,43] (Table 1).

The midpoint between each pair of key landmarks, denoted as p1 and p2 with coordinates x_1_, y_1_, x_2_, and y_2_, was determined using the following formulas:

$$X_{m}=\frac{x_{1}+x_{2}}{2}$$

$$Y_{m}=\frac{y_{1}+y_{2}}{2}$$

Applying these formulas generates secondary landmarks at the midpoint between each pair of primary landmarks. Additionally, tertiary landmarks can be interpolated between the primary and secondary landmarks. This process creates a list of coordinates for these points, facilitating subsequent analysis stages. These points can define various distances, angles, and triangles within the cranial region, allowing for the extraction of individual identification characteristics. The following sections outline the methods for extracting and storing these features.

### 2.3. Geometric Measurement of Craniofacial Bone Structures

To encode the structure of the cranium, it is necessary to calculate various features such as the length, angle, and area of different regions within the cranium, including measurements between all landmarks. Applying these methods resulted in the generation of three lists containing values (Table 2). The output includes three lists of values related to the distance (D), angle (Ag), and area (Ar). The order of items in the lists is predefined and not random.

### 2.4. Ratio Calculation

**Figure 3:**
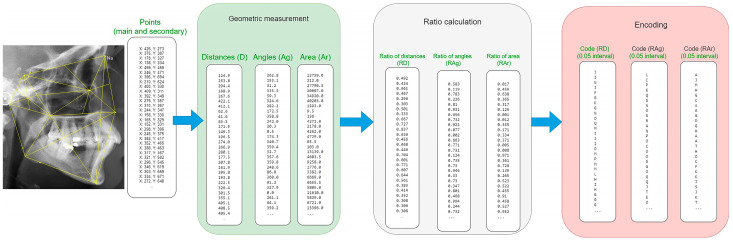
Visual representation of the process of skull pattern-to-string conversion by the algorithm.

To address the differences in magnification between AM and PM radiographs, ratios were used to rescale the values and eliminate magnification discrepancies. Additionally, the encoding process requires that the ratios fall within the range of 0 to 1. In this algorithm, the ratios for the lists D, Ag, and Ar are calculated by dividing each element by every other element within the same list, creating new lists RD, RAg, and RAr with values ranging from 0 to 1 (Figure 3; Table 3). The order of items in these lists is fixed and follows a predefined sequence.

### 2.5. Encoding

In this stage, we used a specific encoding method to represent values between 0 and 1 with intervals of 0.1, 0.05, or 0.02. Table 4 shows the encoding at 0.1 intervals.

The encoding process was conducted as follows:

- Encoder_1 = Code (RD) + Code (RD) + Code (RD × RAg) + Code (RD × RAr).
- Encoder_2 = Code (RAg) + Code (RAg) + Code (RAg × RD) + Code (Rag × RAr).
- Encoder_3 = Code (RAr) + Code (RAr) + Code (RAr × RD) + Code (RAr × RAg).
- Combination (string) = Encoder_1 + Encoder_2 + Encoder_3.

To illustrate Encoder_1, consider an element in the RD list with a value of 0.72, encoded as (H) and duplicated as (HH). Next, we compute the product of each RD element with the corresponding RAg element (RD × RAg), encode the product, and append it to the existing code. For example, if the product is 0.16, it is encoded as (B) and appended to form (HHB). This process is repeated for the products of RD × RAr, with these values also encoded and appended. Encoder_1 processes each value sequentially from the first to the last element in the RD, RAg, and RAr lists. Encoder_2 and Encoder_3 follow a similar approach, resulting in three lists of character strings.

Two regularizers are introduced in this stage:

1. Regularizer 1: This regularizer addresses the algorithm’s sensitivity to less stable cranial structures, such as the mandibular position (lower jaw). The position of the mandible can change over time due to factors like tooth loss, decay, and minor growth during adulthood [46], which can affect its reliability for identity recognition and reduce accuracy. To mitigate this issue, the regularizer assigns greater weight to measurements of more stable structures, such as the maxilla and cranial base, and less weight to the mandible (Figure 2).
2. Regularizer 2: This regularizer defines interval values for encoding, with options for 2%, 5%, or 10% intervals. For example, values can be encoded in 2%, 5%, or 10% intervals, where values from 0 to 0.02 or 0 to 0.05 are assigned a specific code (e.g., code A). The same applies to the 10% interval. By default, a 5% interval is used, balancing between hypersensitivity (2%) and hyposensitivity (10%) in the encoding process.

### 2.6. Storage of Encoded Cephalogram Data

In this algorithm, each individual’s cranial patterns are encoded into character strings and stored in the database along with their corresponding identities. Figure 3 visually illustrates how the K-VIN algorithm converts skull patterns into lists of character strings.

### 2.7. Victim Identification Stage

At this stage of the algorithm, the PM cephalogram is imported, and the user identifies the main landmarks. Secondary landmarks, distances, areas, angles, and their ratios are then automatically generated, encoded, and stored temporarily as victim_encoded. In the next stage, victim_encoded is compared to the stored cases in the database. This comparison is performed in a loop, matching the victim’s encoded values against each database case to identify the case with the highest similarity.

The Levenshtein distance method was utilized to measure the difference between the victim string and the stored strings in the database [47]. This method calculates the minimum number of edits required to transform one string into another. For instance, the Levenshtein distance between “ABCD” and “ABCE” is 1, indicating a similarity score of 75%. A smaller difference indicates a higher similarity between the two strings. The search process continues until the case with the highest level of similarity to the victim code is found in the database.

$$Similarity=1-\frac{Levenshtein\;distance}{\mathrm{Max}\;\left(Length\;of\;source,\;Length\;of\;Target\right)}$$

Once a similar case is identified in the database, the search halts, and the name (and information) of the matching case is output. This case represents the individual most similar to the victim. If the similarity of any case is below the default threshold of 90%, the search continues for a better match. If the similarity exceeds 90%, the Auto Error Reduction (AER) function is activated. The operator can adjust the sensitivity level of this threshold. Additionally, the system can generate a list of similar cases, arranged in descending order of similarity.

It is reported that acceptable accuracy levels for cephalometric landmark selection are 0.59 mm (x coordinate) and 0.56 mm (y coordinate) in total error for diagnostic purposes [48]. Additionally, the reported radius area for the selection landmarks in previous studies was about 2 mm [49]. The Auto Error Reduction (AER) function is designed to minimize user-induced errors in the algorithm. It addresses potential discrepancies between landmarks identified in the PM cephalogram and those in the AM cephalogram of the same individual. Initially, the user selects landmark points from the AM cephalogram, and the algorithm processes these points to encode the individual’s identity in the database. If the same individual later becomes a victim, their PM cephalogram is imported into the software and analyzed by either the same or a different orthodontist. The AER function helps minimize the impact of user errors during the victim analysis phase. If a case in the database shows high similarity but less than 100%, it indicates that the algorithm likely identified the victim correctly. However, the similarity score may fall short of 100% due to potential user errors and variations in landmark selection between the AM and PM radiographs.

**Figure 4:**
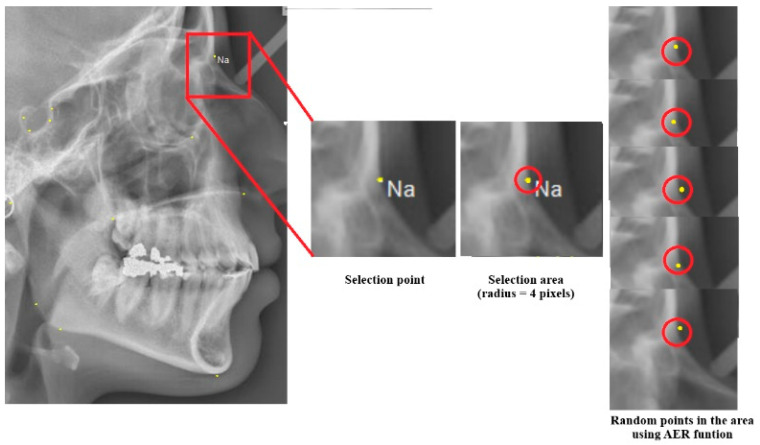
The AER function can generate random points within the landmark area (red circle). This process is applied to the key landmarks to account for potential variations.

In the AER function, a region of interest with a radius of 4 pixels is considered around each key landmark point, rather than focusing on a single point (Figure 4). This approach accounts for the potential variation in landmark placement.

In the AER function, random points are generated within the landmark area, with a default radius of 4 pixels. The algorithm is executed for each set of points to compute the similarity. If the similarity improves, the updated landmark points are stored, and the process continues until the similarity reaches its maximum value. This iterative process is repeated between 100 and 1000 times by default, allowing for potential improvements in the similarity percentage. The radius of the landmark area can be adjusted by the user.

### 2.8. Algorithm Testing (Experimental Setup)

**Figure 5:**
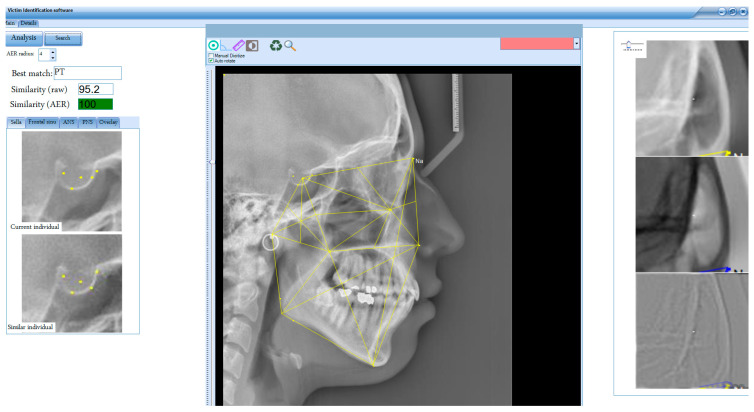
User interface of the software developed in this study. The application automates all stages and processes (excluding key point selection). It provides outputs such as the best match, similarity scores (before and after AER), and a sorted list of the closest matches and displays anatomical landmarks for both the current and similar individuals (e.g., frontal sinus) to verify identity recognition.

A software application was developed using C# in Microsoft Visual Studio 2019 to implement the K-VIN algorithm (Figure 5). Initial testing of the algorithm was conducted using a dataset of 400 pre- and post-treatment digital cephalograms of orthodontic patients (living individuals). Due to the lack of an AM cephalogram archive for deceased individuals, we used cephalograms from an orthodontic archive at a dental clinic. Pre-treatment cephalograms were designated as AM, and post-treatment cephalograms were designated as PM.

Cochran’s formula was used to calculate the sample size, yielding a minimum of 385 samples for 95% confidence and a 5% margin of error. To enhance the precision of the results, 400 samples were considered, each with an AM and a PM image. The samples consisted of orthodontic patients aged 18 and older, each with a minimum treatment duration of 2 years, collected from a dental clinic in Tehran, Iran. Inclusion criteria included the absence of skeletal changes such as maxillofacial surgery and the availability of high-quality pre- and post-treatment cephalometric images. Exclusion criteria comprised a history of jaw trauma during orthodontics, low-quality images, and treatments that caused significant mandibular changes, such as open-bite correction with molar intrusion or maxillary total arch distalization. Only records of patients who underwent orthodontic treatment (extraction or non-extraction) without significant skeletal modifications were included. All cephalometric images were resized to a width of 1000 pixels while maintaining the aspect ratio. The software auto-rotated the images to align the nasion–sella line (cranial base) at a 6-degree angle to the true horizontal. In the software, the process is defined such that the user first selects the nasion and sella points to enable the automatic rotation of the images. This aligns the nasion–sella line (cranial base) at a 6-degree angle to the true horizontal. After this initial selection, the user proceeds to select the remaining landmarks, ensuring consistent image alignment across all cases. The 6-degree adjustment corresponds to the angle between the true horizontal plane and the cranial base in the natural head position [2].

**Figure 6:**
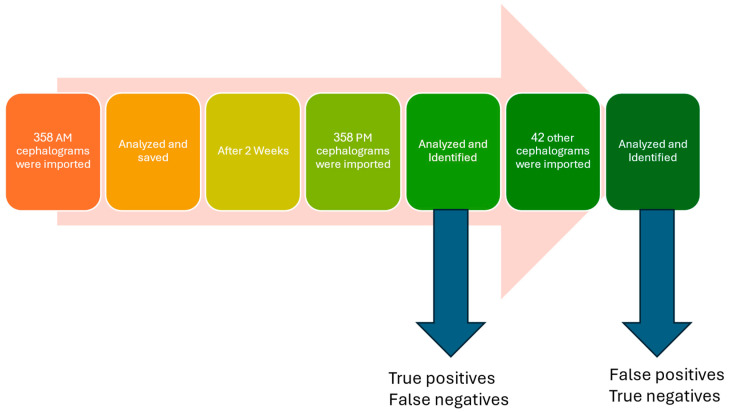
The process of testing the algorithm in this study.

The pre-treatment cephalogram for each patient was labeled as the AM cephalogram, while the post-treatment cephalogram of the same patient was designated as the PM cephalogram. This allowed us to evaluate the software’s performance in identifying individual identities. Figure 6 illustrates the process of testing the algorithm used in this study.

**Figure 7:**
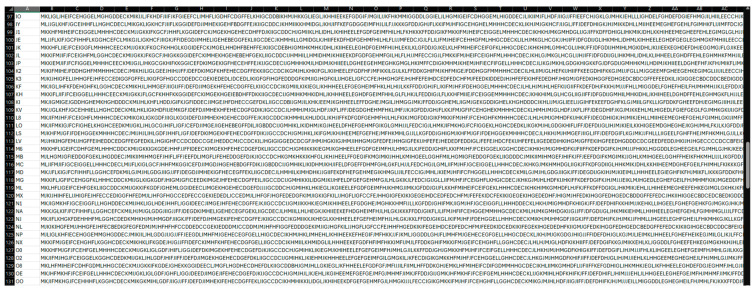
Display of the system database. The first column lists the sample names (AM), and the second column shows the encodings generated by the algorithm.

We imported 358 AM cephalogram images into the software, selected landmarks, and processed them with the algorithm. Each individual’s cephalogram patterns were converted into string representations and stored in the database under their names (Figure 7).

**Figure 8:**
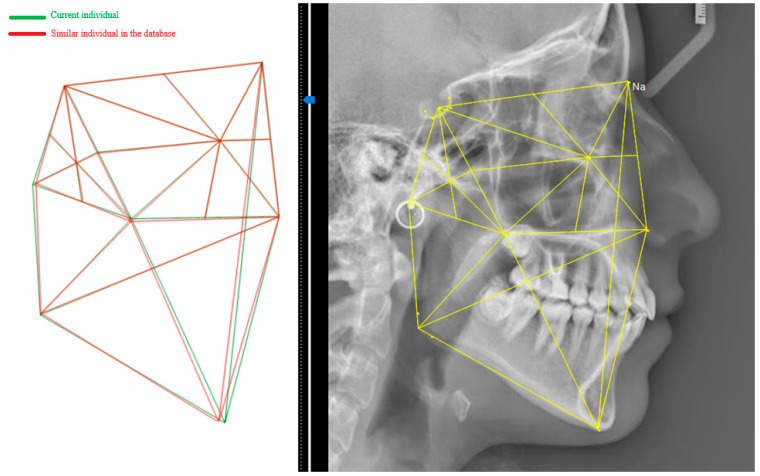
The K-VIN algorithm considers a greater number of ratios in the superior structures than in the inferior structures to minimize the impact of mandibular changes on identity recognition. The superimposition and pattern matching of the current case (green) onto a similar pattern found in the database (red) indicate that the algorithm has successfully performed identity recognition. Despite the similarity in the overall structure, there is a slight difference in mandibular positioning due to orthodontic treatment. It appears that orthodontic treatment has caused a minor change in the lower jaw position.

**Figure 9:**
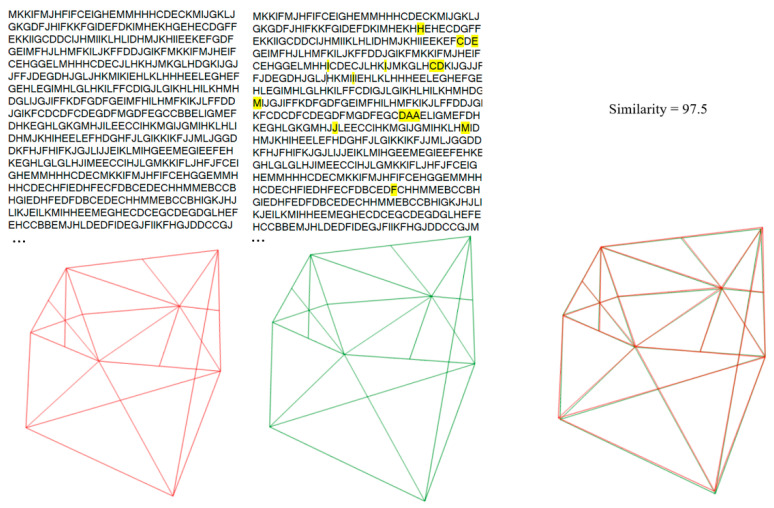
Comparison of the generated string for current individual as PM (green) and the similar individual found in the database as AM (red).

**Figure 10:**
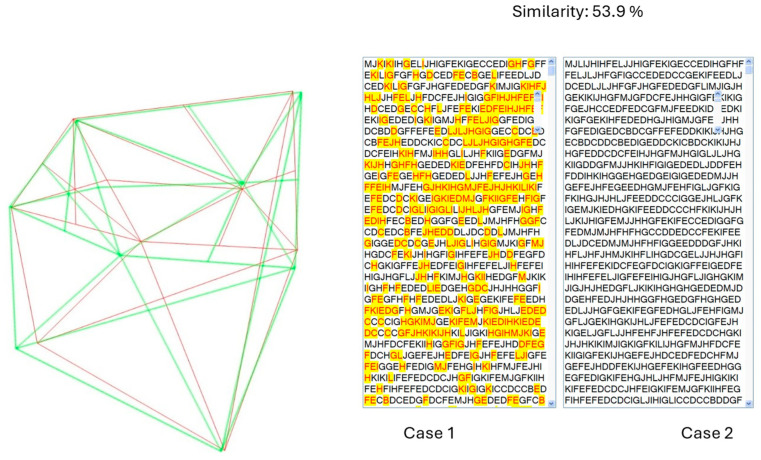
Comparative analysis of cranial patterns for two distinct individuals (non-overlapping).

After a two-week interval, PM cephalograms were imported and processed by the algorithm, recording identity recognition results and similarity percentages. The two-week gap helps prevent potential bias by ensuring that users do not remember specific landmark points from the initial analysis. The algorithm’s output was compared to the actual identities to calculate the accuracy index. To evaluate sensitivity and specificity, 42 cephalograms that were not included in the database were processed. Incorrect matches were recorded as false positives, while correct identifications of no match were recorded as true negatives. Figure 8 shows a sample test demonstrating the alignment of cranial patterns between the AM and PM cephalograms of the same individual. Figure 9 depicts the encoded representations of both the current individual and the matching individual from the database, as identified by the algorithm. Figure 10 illustrates the cranial patterns of two different individuals that do not align.

To assess intra-observer errors in selecting the key landmarks, 20 cephalometric images were randomly selected, and the landmarks were re-identified. The coordinates of each landmark before and after re-identification were compared using Euclidean distance.

## 3. Results

**Figure 11:**
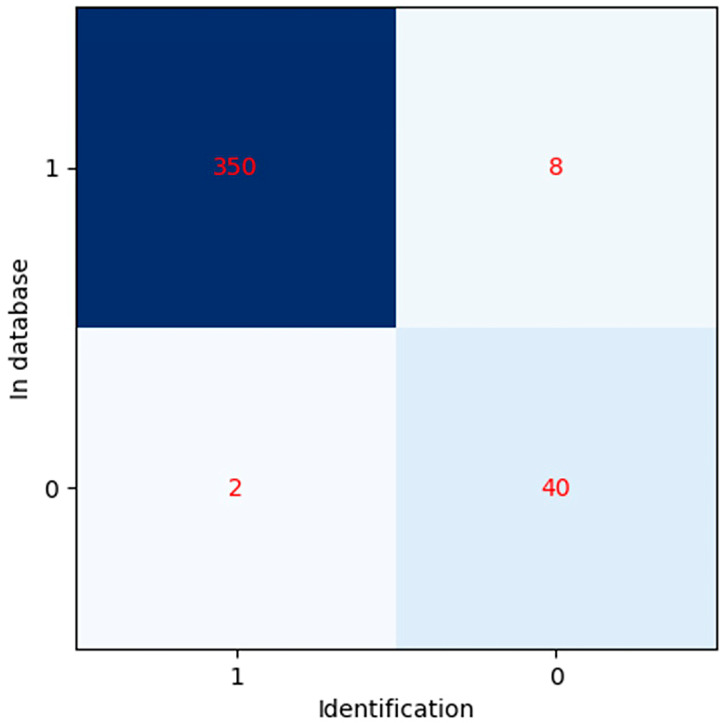
The confusion matrix.

Among the 400 samples analyzed, 48% were female and 52% were male. The average duration between the two cephalograms was 2.58 ± 0.52 years. Table 5 shows the mean and standard deviation for age, initial similarity, and similarity after applying the AER function. A significant correlation was found between raw similarity values for correctly identified cases and those after applying the AER function (*p* < 0.001). The confusion matrix (Figure 11) shows that out of three hundred and fifty-eight cases in the database, three hundred and fifty were accurately identified (true positive), while eight were not (false negative). Out of the forty-two cases not included in the database, forty were correctly identified as not present (true negatives), while two were incorrectly identified as present (false positives). Statistical analysis revealed that the similarity values before and after applying AER did not follow a normal distribution (Kolmogorov–Smirnov test, *p* < 0.05). The Mann–Whitney U test revealed a significant difference between the two distributions (*p* < 0.001).

**Figure 12:**
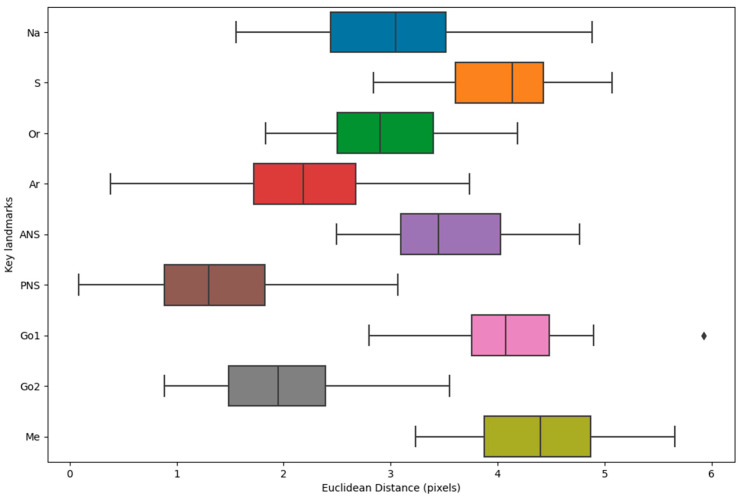
Box plot showing the distribution of Euclidean distances for each landmark in the first and repeated measurements across 20 randomly selected cases.

**Figure 13:**
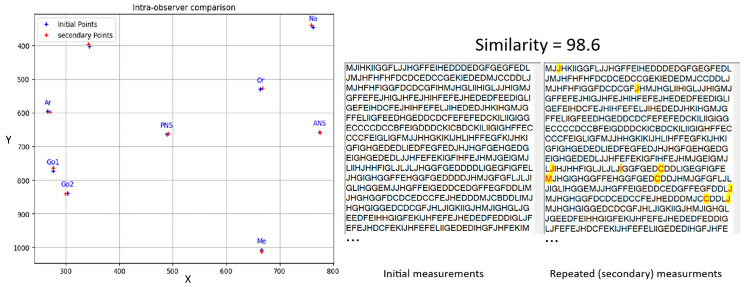
Display of the coordinates of landmarks selected in the first (blue dots) and repeated measurements (red dots) for one of the twenty samples.

The analysis of intra-observer error revealed the following mean Euclidean distances (with standard deviations) between key landmarks in the initial and repeated measurements (Table 6 and Figure 12): Na: 3.07 ± 0.89, S: 4.07 ± 0.61, Or: 2.93 ± 0.65, Ar: 2.23 ± 0.90, ANS: 3.55 ± 0.67, PNS: 1.37 ± 0.75, Go1: 4.09 ± 0.73, Go2: 1.99 ± 0.69, and Me: 4.39 ± 0.69. The Me landmark had the highest mean distance, while the PNS landmark had the lowest. Figure 13 shows the coordinates of points in the initial and repeated measurements for a sample. On average, there was a difference of 1.96 ± 0.99 pixels between points in the two stages, with a 1.4% difference in encodings.

## 4. Discussion

The test results show that the algorithm is highly accurate in identifying individuals based on cranial patterns. Previous studies have explored the use of the frontal sinus, observed through lateral cephalograms [50,51,52], for identity recognition [22,50,53]. However, it should be noted that the accuracy of using the frontal sinus alone may be lower in two-dimensional radiographs than considering the entire cranium. Moreover, the dimensions of the frontal sinus change during growth. A study by Marsya et al. [54] demonstrated that age and gender could be estimated from the frontal sinus, and its growth could be observed until the age of 20. Thus, considering all cranial patterns—not solely the frontal sinus—could enhance accuracy in identity recognition. Similar to fingerprints or facial features, cranial structures may represent a unique identifier for individuals. A review article [55] delves into the possibility of human identification using both two-dimensional and three-dimensional images of the frontal sinus, highlighting the potential role of orthodontists in this field. However, the K-VIN algorithm employs a 2D cephalogram for identity recognition due to its simplicity and cost-effectiveness.

The K-VIN algorithm extracts geometric patterns from the cranium and converts them into character strings to perform identity recognition. It requires an available AM cephalogram for comparison. In situations involving mass casualties where soft tissue is completely lost, PM cephalograms can be obtained to facilitate identity recognition. The application of this algorithm, alongside other identification methods such as odontology [56] and genetics [57], is particularly relevant for high-risk occupations such as firefighting. Organizations, such as firefighting departments, can acquire cephalograms for their personnel. The K-VIN algorithm stores the cranial patterns of individuals in a database, and in the event of a mass casualty incident, a PM cephalogram can be obtained from a body to perform identity recognition based on the algorithm. Identity recognition is performed by identifying the “first matching case found in the database.” In this study, eight cases were incorrectly identified as the “first matching case found in the database”; however, they were the second match in the sorted list. Employing this algorithm allows for identity recognition in mass casualty incidents (given an initial database of individuals), and a sorted list of similarities from the highest to lowest can be generated within the software. The advantage of this method is the speed and cost-effectiveness of the identity verification process. Increasing the computational power of the algorithm, such as by expanding the number and size of ratios and enhancing the generated string, can potentially improve its accuracy by closer to 100%. The proposed method may also be useful to select possible identity suspects to which traditional identification methods can be applied.

In this study, the AER function was used to reduce landmark selection errors during the PM stage. This function automatically adjusts the landmarks to new positions (within a 4-pixel range) to increase the similarity with the AM sample. This adjustment aims to make the similarity measure less dependent on operator error in the PM stage. Although the exact coordinates of points in the AM and PM samples are not identical (due to potential differences in aspect ratio), AER modifies the PM coordinates to enhance similarity, within a 4-pixel range for each landmark. In this study, intra-observer error revealed that, on average, the Euclidean distance (in pixels) between repeated measurements for an observer was approximately 3.07 with a standard deviation of 0.73 pixels. This finding is consistent with the study by Hägg et al. [49]. However, future studies could fully automate the landmark selection process using artificial intelligence algorithms, such as key point detection models like Mediapipe, which is used for facial landmark detection in identity recognition [58].

Since the proposed method is metric-based, it is possible for multiple individuals to have relatively similar measurements. However, based on the results of this study, it appears that combining various geometric features extensively can enhance individual differences and increase the similarity of the same individual across different times. Additionally, the algorithm can generate a list of similar cases found in the database for identity screening. It has also been reported that the geometric pattern of cranial components varies among individuals [59,60,61]. This algorithm anticipates that the position of the mandible may change over time, thereby assigning less weight to the mandible’s features in the calculations. A study by Patil et al., which focused on personal identification in 100 living individuals with an average age of 25, showed that the frontal sinuses are unique to each person [52]. In this study, with a sample of 400 patients, we found that the overall cranial structure appears to be nearly unique for each individual. However, further research on cadavers is needed. One limitation of this algorithm is its reduced accuracy in cases where the patient’s jaw has been affected by skeletal fractures and displacements. Future studies should aim to enhance the algorithm’s ability to handle such cases.

The algorithm has several limitations. It struggles with identifying cases involving severe maxillofacial trauma. To address this, generating a virtual reconstructed view of the lateral cephalogram using advanced AI techniques might enhance the algorithm’s performance in such cases. However, it is important to emphasize that this is a foundational algorithm with substantial potential for further development and refinement. Another limitation is the lack of testing on deceased individuals due to the unavailability of antemortem cephalograms. Therefore, pre- and post-treatment images from an orthodontic archive were used for testing. Additionally, the accuracy of landmark selection depends on user input, which could be improved by incorporating AI methods such as key point detection [62]. In the field of dental and maxillofacial diagnostics, AI has demonstrated substantial progress in automating the analysis of cephalometric data [63]. AI algorithms are now used to automatically detect and measure cephalometric landmarks, which are crucial for diagnosing orthodontic conditions and orthognathic surgery. By employing AI techniques, it would be possible to consider more key landmarks, leading to more detailed feature calculations and improving the overall accuracy of the algorithm. A proposed design for future works could involve using advanced machine learning models, such as convolutional neural networks (CNNs), specifically trained on large datasets of cephalometric images to identify and classify key landmarks with high precision. While there are advanced AI systems in medical imaging and diagnostics, few directly address the forensic application of cranial pattern recognition from lateral cephalometric images. More recently, CNNs have shown promise in classifying sagittal skeletal patterns, with DenseNet161 achieving the highest accuracy [64]. Clustering techniques have also been effective in identifying craniofacial morphological patterns using multivariate cephalometric data [65]. However, despite these advancements, the application of AI in forensic identification using cephalometric radiographs remains largely unexplored. This gap presents an intriguing research opportunity to integrate these emerging AI techniques with our algorithm, which could advance identity verification methods through cranial pattern analysis.

Despite these limitations, the algorithm shows considerable potential for further development and integration with AI technologies. Future studies should validate the algorithm with multiple bodies using available antemortem and postmortem cephalograms. Incorporating AI for automatic landmark identification could enhance the algorithm’s framework. Comparing the K-VIN algorithm with other identification methods will help understand its performance and strengths better. This algorithm may have applications in forensic identification, orthodontics, and anthropological research, aiding in identifying missing persons, evaluating treatment outcomes, and analyzing craniofacial variations. It could also serve as a foundation for advancements in imaging and analysis technologies, particularly when integrated with AI techniques.

## 5. Conclusions

In conclusion, the K-VIN algorithm demonstrates exceptional accuracy, sensitivity, and specificity, making it a highly promising tool for forensic medicine. By analyzing cranial patterns from lateral cephalometric radiographs, the algorithm improves upon existing methods that often rely solely on frontal sinus features. It encodes and compares geometric patterns from AM and PM images for identification. Despite its promising results, challenges such as handling severe trauma cases and variability in manual landmark selection remain. Future research should focus on integrating automated landmark selection through advanced AI techniques, such as key point detection, to further refine the algorithm’s accuracy and efficiency. Testing on deceased individuals and expanding automated processes will be crucial for realizing the algorithm’s full potential.

## 6. Patents

The author S.K. is the inventor of the K_VIN algorithm, which is covered by Iranian Patent No. 109874. This patent is registered with the Iranian Patent Office. For more details, see the official patent record at the Iranian Patent Office (https://ipm.ssaa.ir/Search-Result?page=1&DecNo=140250140003000674&RN=109874, accessed on 15 August 2024).

## Figures and Tables

**Table 1:** The key landmarks in the lateral cephalometry used for this study.

Landmark	Description
Nasion (NA)	The anterior point where the nasal and frontal bones intersect [42,43].
Sella (S)	The midpoint of the pituitary fossa, also known as the sella turcica [42,43].
Orbitale (Or)	The lowest point on the inferior margin of the orbit [42,43].
ANS	Anterior nasal spine, the tip of the anterior nasal spine (sometimes modified as the point on the upper or lower contour of the spine where it is 3 mm thick) [42,43].
PNS	The posterior nasal spine, defined as the tip of the palatine bone’s posterior spine at the junction between the hard and soft palates [42,43].
Articulare (Ar)	The point where the contour of the posterior surface of the mandibular condyle intersects with the temporal bone [42,43].
Gonion (Go)	The midpoint of the contour connecting the most inferior point of ramus (Go1) and the most posterior body of the mandible (Go2) [44]. The midpoint in the mediolateral dimension on the most posterior border of the mandible [42,43].
Menton (Me)	The most inferior point on the chin [42,43].

**Table 2:** Geometric calculations of different regions within the cranium, between landmarks.

Parameter	Definition	Formula
Distance (D)	The Euclidean distance between each pair of landmarks, denoted as p1 and p2, respectively. Output (list of values): D = {D_1_, D_2_, …, D_n_}	D = $\sqrt{{\left(p2.X-p1.X\right)}^{2}+{\left(p2.Y-p1.Y\right)}^{2}}$
Angle (θ)	The angle between every set of three landmarks (p1, p2, and p3) with coordinates (x1, y1), (x2, y2), and (x3, y3) can be calculated by the following: First, vectors A and B are defined; then, their lengths are computed, followed by calculating their dot product, and finally, the angle (θ) between the three landmarks is obtained. Output (list of values): Ag = {θ_1_, θ_2_, …, θ_n_}	$A=\left(x_{1}-x_{2},y_{1}-y_{2}\right)$ $B=(x_{3}-x_{2},y_{3}-y_{2})$ $|A|=\sqrt{(x_{1}-x_{2}{)}^{2}+(y_{1}-y_{2}{)}^{2}}$ $|B|=\sqrt{(x_{3}-x_{2}{)}^{2}+(y_{3}-y_{2}{)}^{2}}$ $A\cdot B=\left(x_{1}-x_{2}\right)\left(x_{3}-x_{2}\right)+\left(y_{1}-y_{2}\right)\left(y_{3}-y_{2}\right)$ $\theta =\frac{180}{\pi }{cos\;}^{-1}(\frac{A\cdot B}{|A||B|})$
Area (Ar)	The area of each triangle by each set of three landmarks can be determined using Heron’s formula [45]. To calculate the area of a triangle given the lengths of its sides (a, b, and c), first, the semi-perimeter (s) is defined; then, Heron’s formula is used to determine the area. Output (list of values): Ar = {Ar_1_, Ar_2_, …, Ar_n_}	$s=\frac{a+b+c}{2}$ $Ar=\sqrt{s(s-a)(s-b)(s-c)}$

**Table 3:** Ratio calculations.

Parameter	Definition	Formula	Output (List of Values)
RD	The ratio of each line segment’s length to another was calculated and constrained between 0 and 1 by always dividing the smaller length by the larger length.	$RD=\frac{min\;(a,b)}{max\;(a,b)}$	RD = {a_1_, a_2_, …, a_n_} 0 < a <= 1
RAg	The ratio of each angle to another angle was calculated to obtain this index, ensuring values between 0 and 1 by always dividing the smaller angle by the larger angle.	$RAg=\frac{min\;(a,b)}{max\;(a,b)}$	RAg = {b_1_, b_2_, …, b_n_} 0 < b <= 1
RAr	The ratio of each triangle’s area to another triangle’s area was computed, ensuring values between 0 and 1 by always dividing the smaller area by the larger area.	$RAr=\frac{min\;(a,b)}{max\;(a,b)}$	RAr = {c_1_, c_2_, …, c_n_} 0 < c <= 1

**Table 4:** The encoding scheme for values ranging from 0 to 1 (0.1 interval).

Value	Code
0–0.1	A
0.1–0.2	B
0.2–0.3	C
0.3–0.4	D
0.4–0.5	E
0.5–0.6	F
0.6–0.7	G
0.7–0.8	H
0.8–0.9	I
0.9–1	J

**Table 5:** Algorithm performance metrics on the test data, including similarity, accuracy, sensitivity, and specificity.

Parameter	Definition	Result
Age (years)	Mean age ± standard deviation (SD) of samples	22.21 ± 4.5
Similarity	Mean similarity ± SD	91.02 ± 2.6%
Similarity_AER	Mean similarity ± SD after applying the AER function	98.10 ± 3.37%
Accuracy	(TP + TN)/(TP + TN + FP + FN)	0.975
Sensitivity	TP/(TP + FN)	0.977
Specificity	TN/(TN + FP)	0.952

**Table 6:** Euclidean distances between the landmarks from the first and repeated measurements in the intra-observer assessment for twenty randomly selected cephalometric samples.

	Na	S	Or	Ar	ANS	PNS	Go1	Go2	Me
1	4.233824	4.241515	3.035855	1.607974	2.95384	1.104472	4.896409	0.8858	4.854231
2	3.041754	4.631722	2.73602	3.530173	3.316864	1.459843	3.550859	0.966853	4.613667
3	3.279826	4.121985	2.573237	1.756341	4.302755	0.0854	4.44715	2.540002	4.421004
4	3.685795	4.362348	2.158848	1.821404	4.142899	2.22766	3.914375	1.915114	3.360346
5	3.045192	3.952148	2.215952	2.259347	3.713335	2.177729	4.085907	1.808773	3.816824
6	2.013631	4.297177	3.405217	0.985085	3.595169	1.449267	4.303165	1.838328	5.654552
7	4.738587	4.166425	3.456717	1.496716	4.764213	0.534224	4.581135	1.980466	4.912953
8	2.89302	4.155614	2.137153	3.506202	4.763274	0.514616	3.138656	2.695381	4.352564
9	1.551962	3.596292	2.295773	2.629039	2.685305	2.10584	4.133792	2.322259	3.718152
10	3.614392	3.609049	4.107028	3.739961	3.343799	0.92952	5.922959	2.687414	3.890276
11	2.1054	3.300165	3.393195	1.387695	3.034395	1.733976	4.372208	1.746393	3.356147
12	1.913625	4.078769	2.612472	2.188649	3.986002	1.062368	2.900914	1.117798	4.692608
13	2.268908	3.204426	4.185395	2.02836	3.406368	1.14404	4.631237	1.294914	3.921766
14	3.17777	3.490724	3.138276	1.924587	3.488155	2.142705	3.980733	2.246511	4.373506
15	3.074464	4.85546	3.241534	3.69165	3.600555	1.556844	3.824773	1.272621	4.184124
16	3.478464	2.838455	1.829814	2.50061	3.110236	0.745751	4.058575	2.336319	5.099332
17	2.835942	4.613818	3.104121	2.795506	2.58939	0.645531	3.949099	1.552377	3.230014
18	4.883149	5.029736	3.564132	0.380446	3.33089	3.066221	2.801093	3.5481	4.848982
19	2.494161	5.070583	2.616539	2.177864	2.492837	1.143635	4.83874	2.328205	5.04226
20	2.979627	3.883471	2.772158	2.207089	4.396735	1.729498	3.392602	2.778084	5.392706

## Data Availability

The source code for this project (and algorithm) is available on GitHub and can be shared upon request.
